# Sensitive and Specific Detection of Estrogens Featuring Doped Silicon Nanowire Arrays

**DOI:** 10.1021/acsomega.1c00210

**Published:** 2022-12-06

**Authors:** Wenqi Duan, Hui Zhi, Daniel W. Keefe, Bingtao Gao, Gregory H. LeFevre, Fatima Toor

**Affiliations:** † Department of Electrical and Computer Engineering, University of Iowa, 205 North Madison Street, Iowa City, Iowa 52242, United States; ‡ Department of Civil and Environmental Engineering, University of Iowa, 4105 Seamans Center, Iowa City, Iowa 52242, United States; § IIHR−Hydroscience & Engineering, 100 C. Maxwell Stanley Hydraulics Laboratory, Iowa City, Iowa 52242, United States; ∥ Iowa Technology Institute, University of Iowa, 330 South Madison Street, Iowa City, Iowa 52242, United States

## Abstract

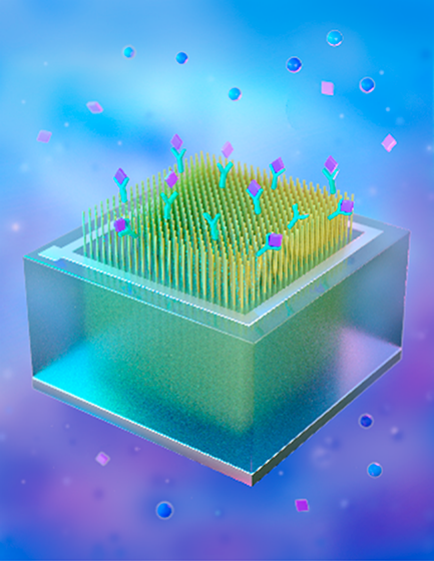

Estrogens and estrogen-mimicking compounds in the aquatic environment are known to cause negative impacts to both ecosystems and human health. In this initial proof-of-principle study, we developed a novel vertically oriented silicon nanowire (vSiNW) array-based biosensor for low-cost, highly sensitive and selective detection of estrogens. The vSiNW arrays were formed using an inexpensive and scalable metal-assisted chemical etching (MACE) process. A vSiNW array-based p–n junction diode (vSiNW-diode) transducer design for the biosensor was used and functionalized via 3-aminopropyltriethoxysilane (APTES)-based silane chemistry to bond estrogen receptor-alpha (ER-α) to the surface of the vSiNWs. Following receptor conjugation, the biosensors were exposed to increasing concentrations of estradiol (E2), resulting in a well-calibrated sensor response (*R*^2^ ≥ 0.84, 1–100 ng/mL concentration range). Fluorescence measurements quantified the distribution of estrogen receptors across the vSiNW array compared to planar Si, indicating an average of 7 times higher receptor presence on the vSiNW array surface. We tested the biosensor’s target selectivity by comparing it to another estrogen (estrone [E1]) and an androgen (testosterone), where we measured a high positive electrical biosensor response after E1 exposure and a minimal response after testosterone. The regeneration capacity of the biosensor was tested following three successive rinses with phosphate buffer solution (PBS) between hormone exposure. Traditional horizontally oriented Si NW field effect transistor (hSiNW-FET)-based biosensors report electrical current changes at the nanoampere (nA) level that require bulky and expensive measurement equipment making them unsuitable for field measurements, whereas the reported vSiNW-diode biosensor exhibits current changes in the microampere (μA) range, demonstrating up to 100-fold electrical signal amplification, thus enabling sensor signal measurement using inexpensive electronics. The highly sensitive and specific vSiNW-diode biosensor developed here will enable the creation of low-cost, portable, field-deployable biosensors that can detect estrogenic compounds in waterways in real-time.

## Introduction

The presence of estrogens and natural and synthetic estrogen-mimicking compounds in aquatic environments is deleterious to ecosystems and human health and has become an emerging water quality concern.^1,2^ For example, the sex distribution of fish can be skewed female downstream from wastewater treatment plant outfalls, and the formation of intersex individuals (possessing both male and female reproductive organs) occurs due to the presence of estrogen-mimicking compounds.^2,3^ Despite the potential ecological impacts, estrogens in the aquatic environment are currently evaluated in a haphazard manner. Individual estrogens can be measured by standard chemical analyses (e.g., mass spectrometry), if the compound is known and characterized *a priori*.^4^ Nevertheless, biologically active metabolites or emerging contaminants can be “masked” during chemical analysis,^5^ thus underestimating the impacts to organisms compared to a receptor-binding perspective. A number of *in vitro* assays, such as those based on enzyme-linked immunosorbent assays (ELISA) and the recombinant yeast estrogen receptor binding assays (YES),^6^ have been developed as an alternative to chemical analysis to measure the estrogenic activity of unknown, complex mixtures relative to 17β-estradiol (E2), which is the most potent natural estrogen *in vivo*.^7^ Unfortunately, *in vitro* assays are laborious, expensive, and incapable of providing real-time information under dynamic environmental conditions. Bioassays involving live organisms are a gold-standard in ecotoxicology but are slow and expensive, do not provide real-time responses, can raise ethical concerns, and may not well represent effects to other organisms.^8^ Chromatographic techniques, such as liquid or gas chromatography–mass spectrometry (LC or GC–MS),^9^ while being highly sensitive and accurate quantitatively, have shortcomings, such as the needed analytical instruments being expensive, a large number of samples and pretreatments and much organic solvent being required, and trained personnel being required to operate the complex systems. Notably, none of these techniques allow for real-time *in vivo* monitoring of waterways. Therefore, the current paradigm for assessing estrogens in aquatic systems is inadequate to protect water supplies and human exposure in drinking water. Thus, there is a critical need to develop new cost-effective technologies and tools, such as biosensors, that enable rapid, sensitive, and selective detection of estrogens in water.

Nanomaterials-based biosensors for water quality sensing have recently become an active area of research.^10^ Nanotechnology-based biosensors offer advantages over conventional analytical techniques, including miniaturization, high specificity for real-time analysis in complex mixtures, high sensitivity, simple operation without extensive sample pretreatment, and low cost. Nanowire (NW)-based materials have a high surface area to volume ratio, thus improving sensitivity. The use of silicon (Si) NW field effect transistor (FET) (SiNW-FET)-based biosensors was first introduced in 2001^11^ and has since been further developed by numerous research groups.^12^ In these works, 1–10 horizontally oriented Si NWs (hSiNWs) are biofunctionalized with receptors^13^ and electrically probed using the FET device structure, which typically requires two electrical contacts: source and drain on each end of the hSiNW. This device design constraint (i.e., electrical source and drain contacts) results in a complex and expensive nanofabrication process, thereby limiting the number of NWs per device.^13,14^ Due to the small number of hSiNWs in the hSiNW-FET biosensors, electrical current change as a function of target-compound concentration is on the order of nanoamperes (nA);^15^ such a small electrical change current response requires expensive and bulky measurement equipment that is not suitable for scalable field deployment.

In this work, we present initial results on a novel vertically oriented Si NW (vSiNW)-based p–n junction diode device architecture (vSiNW-diode) biosensor prototype, which can be developed into a portable sensor system suitable for field measurements of estrogens in waterways. The goal of this initial proof-of-principle study is to demonstrate the vSiNW-diode biosensor’s feasibility in a controlled lab environment. Our biosensor design features an array consisting of millions of n-doped vSiNWs, all electrically contacted to each other using a single metal contact. Each of the vSiNWs in the array is functionalized with the hormone receptor and acts as an anchor for the target hormone species. This vSiNW-diode biosensor design results in much higher electrical current change [on the order of hundreds of microamperes (μA), which can be measured using inexpensive equipment], making our biosensor suitable for real-time highly sensitive and specific field measurements. An additional advantage of the presented vSiNW-diode biosensor is that possible biofouling that could occur on *in vivo* water monitoring sensors will likely be minimized because NWs can reduce fouling coverage by up to ∼60% mainly due to two geometric effects: reduced effective settlement area and mechanical cell penetration.^16^

## Experimental Section

We employ standard complementary metal–oxide–semiconductor (CMOS)-compatible microfabrication process steps, such as high-temperature doping of the front and back of monocrystalline Si wafers, dry etching for edge isolation, photolithography and e-beam evaporation for front metal contact patterning, and sputtering of the dielectric films for front contact protection, to manufacture our biosensors. For the Si NW array biofunctionalization, we applied 3-aminopropyltriethoxysilane (APTES)-based wet-chemistry, demonstrated by other research groups^17^ to successfully attach estrogen receptor-alpha (ER-α) to the surface of the Si NWs. We then measured fluorescence intensity and *J*–*V* response to quantify the biosensor. Details of the microfabrication, biofunctionalization, and testing are presented in the Supporting Information, Sections S.1–S.3. Next, we present a brief summary of the microfabrication and biofunctionalization of our vSiNW-diode sensors.

**Figure 1:**
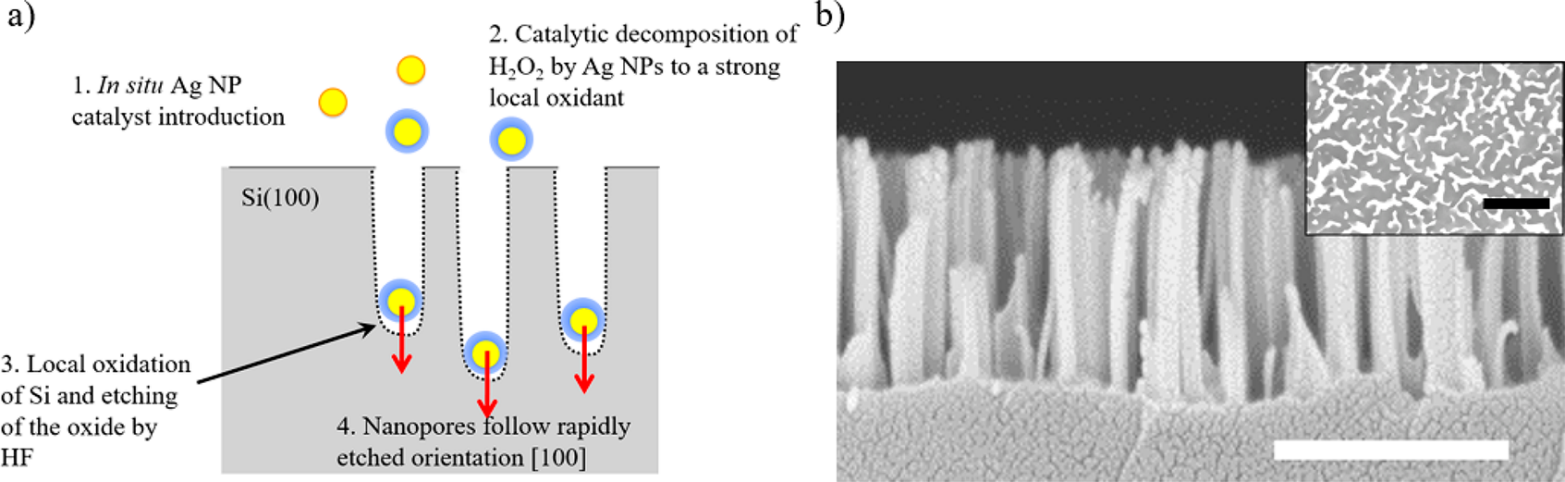
(a) Schematic of the MACE process that results in vertically oriented arrays of Si NWs on the surface of a Si(001) substrate. (b) Cross-sectional and top-view (inset) scanning electron microscopy (SEM) images of Si NW arrays fabricated using the MACE process. Scale bars in the images represent 500 nm.

The vSiNW array in our biosensor is fabricated using metal-assisted chemical etching (MACE)^18,19^ (Figure 1a) where a metal salt (here, silver nitrate [AgNO_3_]) is reduced by hydrofluoric acid (HF) into silver (Ag) nanoparticles (NPs) that then locally catalyze the oxidation of Si into silicon dioxide (SiO_*x*_) in the presence of an oxidant (here, hydrogen peroxide [H_2_O_2_]). The scanning electron microscopy (SEM) images of the resulting vSiNW array (Figure 1b) confirm that the average length of the NWs is ∼500 nm. Our recent works^18−22^ extensively characterize the MACE-generated vSiNWs and, for example, confirm the reproducibility of the NW lengths from the same MACE recipe batch to batch^19^ and the vSiNW-diode architecture as an effective biosensor transducer.^21,22^

**Figure 2:**
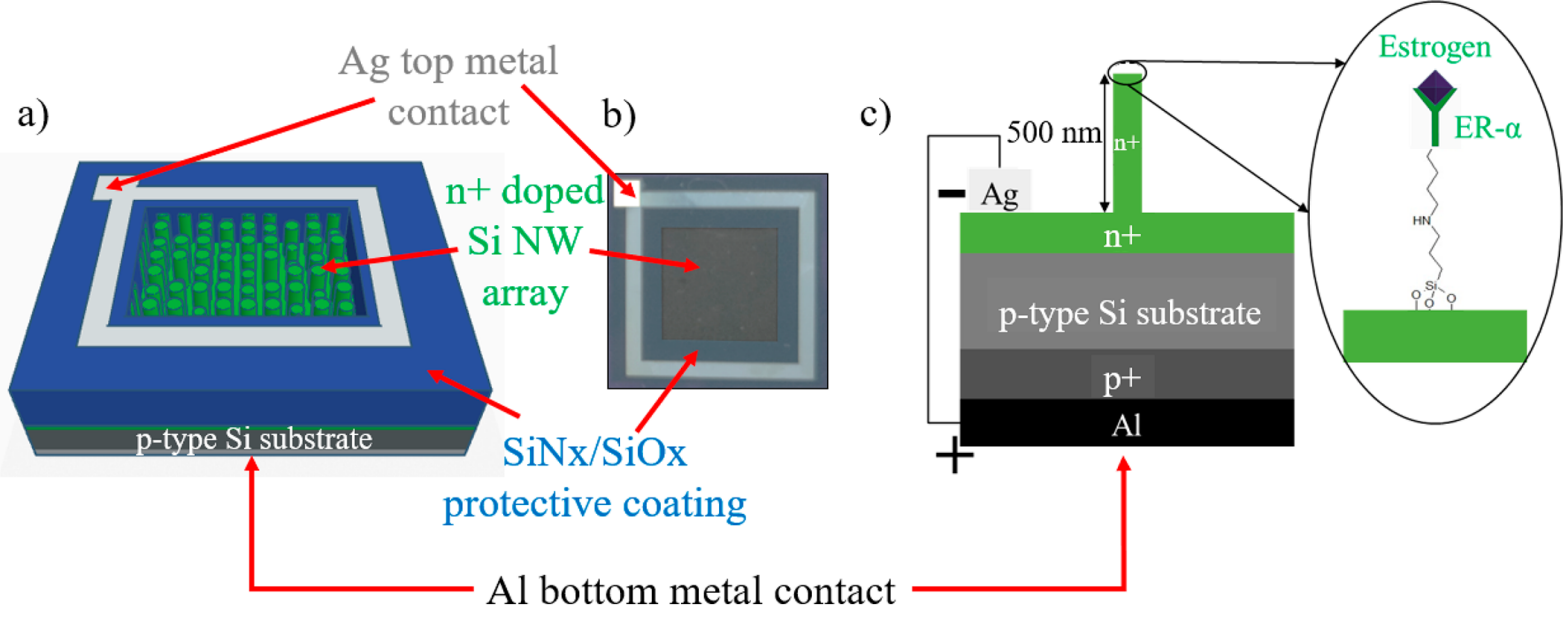
(a) Schematic (not to scale) of the Si NW biosensor showing the key components of the p–n junction device, including the n+ doped Si NWs that are around 500 nm long, Ag top metal contact electrically connecting all the NWs, SiN_*x*_/SiO_*x*_ dielectric stack that protects the metal contact from degrading during biofunctionalization steps, p-type Si substrate that is around 280 μm thick, and Al bottom contact. (B) Optical image of the top-view of the fabricated NW biosensor. The total sensor area is 11.5 mm by 11.5 mm, and after the SiN_*x*_/SiO_*x*_ protective coating on the top metal contact, the exposed NW area is 6.2 mm by 6.2 mm. (C) Enlarged detail schematic (not to scale) of the cross-section of the biosensor showing the various doped regions, metal contacts, and ER-α functionalized NW surface. ER-α will become functionalized not only on the tip of the vSiNWs but also across the entire length of the vSiNWs. The schematic is simplified for clarity.

The nanofabrication process steps for our vSiNW biosensor are inexpensive and scalable and are detailed in the Supporting Information, Section S.2. The electrical design of our vSiNW biosensor employs a p–n junction as its transducing element, resulting in a vSiNW-diode (Figure 2). A p-type Si wafer is used as the substrate on which ∼500 nm long vSiNWs are etched using the MACE process, which are then doped n+ using phosphorus doping, to form an n+ emitter. Based on our previous reported work^20^ for which we used the same ammonium dihydrogen phosphate (ADP)-based proximity doping process, the junction depth achieved is around 700 nm based on secondary mass ion spectroscopy (SIMS) analysis. This junction depth ensures that the vSiNWs are not depleted of carriers. A patterned top metal contact based on 50 nm thick titanium (Ti) and 1 μm thick Ag is deposited to electrically connect to the top vSiNWs array. The 50 nm thick Ti is deposited to improve the adhesion of the 1 μm Ag top metal contact. Ti is commonly used as an adhesion layer for low-contact-resistance metal contacts on silicon-based (opto)electronic devices.^23^ This Ti/Ag metal contact is then covered with a silicon nitride/silicon oxide (SiN_*x*_/SiO_*x*_)-based dielectric stack to eliminate degradation during wet biofunctionalization steps. A p+ back surface field (BSF) and the bottom contact are formed by painting and annealing with aluminum (Al) paste. The top-view of the fully fabricated biosensor is shown in Figure 2b, with a total sensor area of 11.5 by 11.5 mm while the exposed vSiNW area is decreased to 6.2 by 6.2 mm after the SiN_*x*_/SiO_*x*_ protective dielectric stack is deposited on the top metal contact to reduce contact degradation through extensive fluid exposure during biofunctionalization.

After the vSiNW-diode sensor is microfabricated, it is biofunctionalized (described in detail in the Supporting Information, Section S.2) with APTES-based silane chemistry followed by glutaraldehyde, which is a bifunctional linker containing two aldehyde terminals, which enables one end to bind to the amine-terminated APTES and the other end to immobilize the ER-α protein. The ER-α protein is covalently bound onto the surface of the Si NWs, unbound ER-α protein removed with a 0.01× phosphate buffer solution (PBS) buffer wash and passivated with ethanolamine to minimize nonspecific binding. Next, the sensors are tested with the hormones of interest. The current density–voltage (*J*–*V*) electrical measurements are performed immediately following when the estrogen receptor is bound on the Si NW surface; this measurement serves as the tare. Next, target hormones, such as estrone (E1), 17β-estradiol (E2), and testosterone (Figure S3), are exposed on the sensor surface, and the subsequent change in electrical current is measured and quantified. Figure 2c shows an enlarged schematic of the p–n junction biosensor with the receptor bound on the surface (more detailed biofunctionalization process steps in Figure S2).

In this work, we develop an initial prototype of the vSiNW-diode biosensor and demonstrate that vSiNWs yield a significantly higher fluorescence signal than a planar surface, indicating a higher number of estrogen receptors present on the vSiNWs relative to an untextured planar Si surface. We also confirm the biosensor sensitivity using an estrogen concentration dependent *J*–*V* response. Furthermore, we probe the impact of the doping density of the Si NWs on the biosensor response. We test the biosensor selectivity response by comparing it between an estrogen and an androgen. Finally, sensor regeneration tests demonstrate reusability and potential for development of our vSiNW-diode biosensor for field deployment.

## Results and Discussion

### Fluorescence Measurements

**Figure 3:**
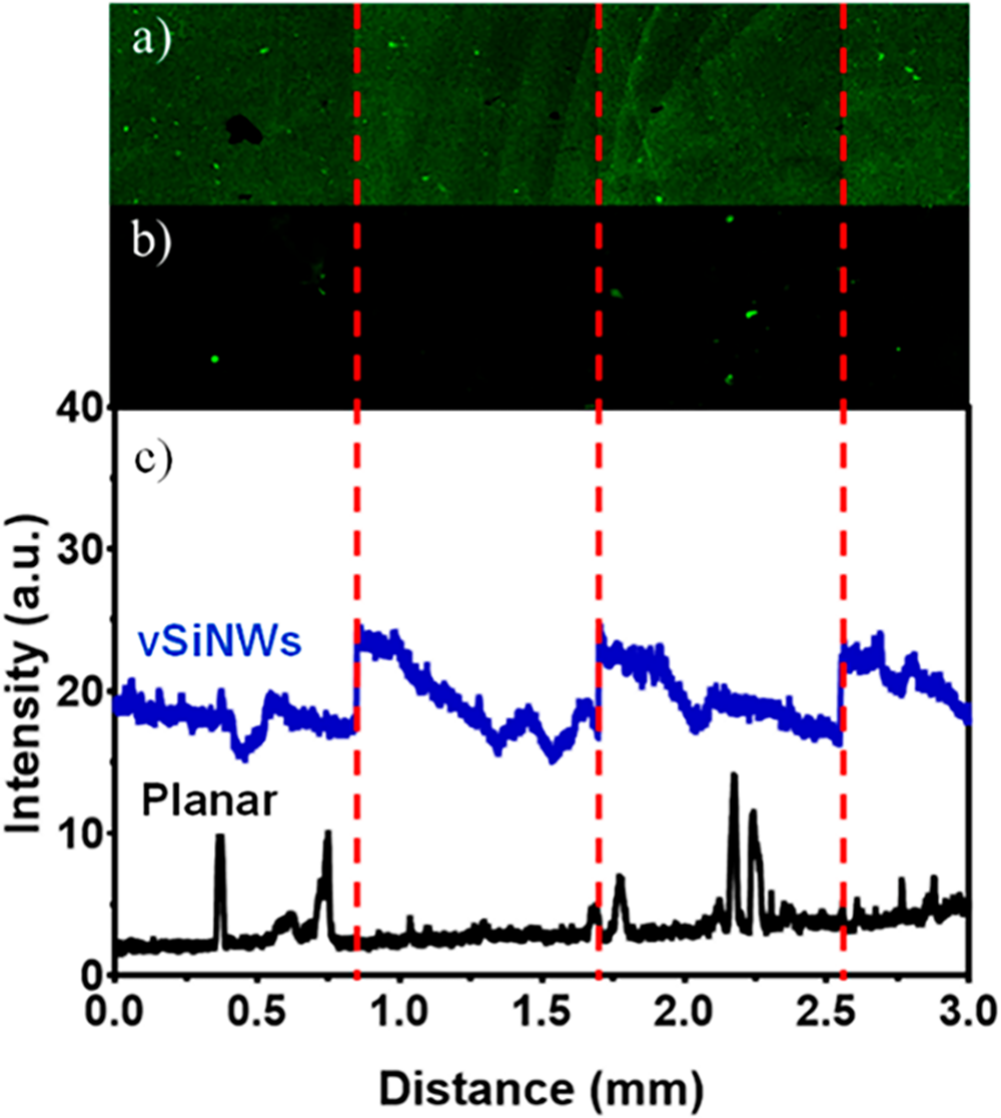
Fluorescence images of (a) a vSiNW surface postfunctionalization and (b) a planar surface postfunctionalization. (c) Visual comparison of the intensities, demonstrating the increased number of receptors present on the Si NW surface. Both the planar and Si NW samples were functionalized with ER-α and stained by fluorescent-tagged antibodies. Each sample was imaged over a 3 mm long by 0.85 mm high area to ensure that the increase in intensity was consistent across a large distance. The red dashed line shows the edges of the tiles that were joined.

Fluorescence measurements confirm that vSiNWs amplify the sensor signal relative to planar biosensors (Figure 3). The details of the preparation of the sensor surfaces for these measurements are provided in the Supporting Information, Section S.3.3. We analyzed the fluorescence images of the vSiNW surface (Figure 3a) and planar surface (Figure 3b) using ImageJ software and plotted the image analysis results (Figure 3c), which indicate that the vSiNWs exhibited a between 4 and 10 times brighter fluorescence signal than the planar Si surface. The brighter intensity fluorescence signal for the NWs confirms that the nanostructured Si surface conjugates a higher concentration of the hormone receptor than the planar surface due to the increased surface area of the NWs and can consequently result in a greater electrical signal change for the same concentration of target analyte. In our recent work,^22^ we indeed confirmed this advantage of vSiNW-diode over planar-diode biosensors, where we reported that vSiNW biosensors exhibit an around 20% relative current density change as compared to a 5% change in planar sensors functionalized with the same concentration of a cancer antigen. These results confirm the advantage of using vSiNWs as a biosensor surface.

### NW Array Doping Density and Biosensor Sensitivity

We fabricated the biosensors with varied doping levels and measured the NW sensor response to a range of E2 concentrations (1, 10, and 100 ng/mL). The biosensor had the largest current density response when the NW array doping was low, which resulted in effective sheet resistivity (*R*_sheet_) values in the 1000–1200 Ω/sq range. Doping levels are directly related to sheet resistivity, where a high *R*_sheet_ value corresponds to a low doping concentration, and vice versa. The effects of both E2 concentration and NW array *R*_sheet_ were evaluated by the change in biosensor current density response. We also compared the current density change of our vSiNW array biosensors and the conventional hSiNW biosensors to demonstrate up to 100-fold electrical signal amplification in our biosensors relative to the conventional ones.

**Figure 4:**
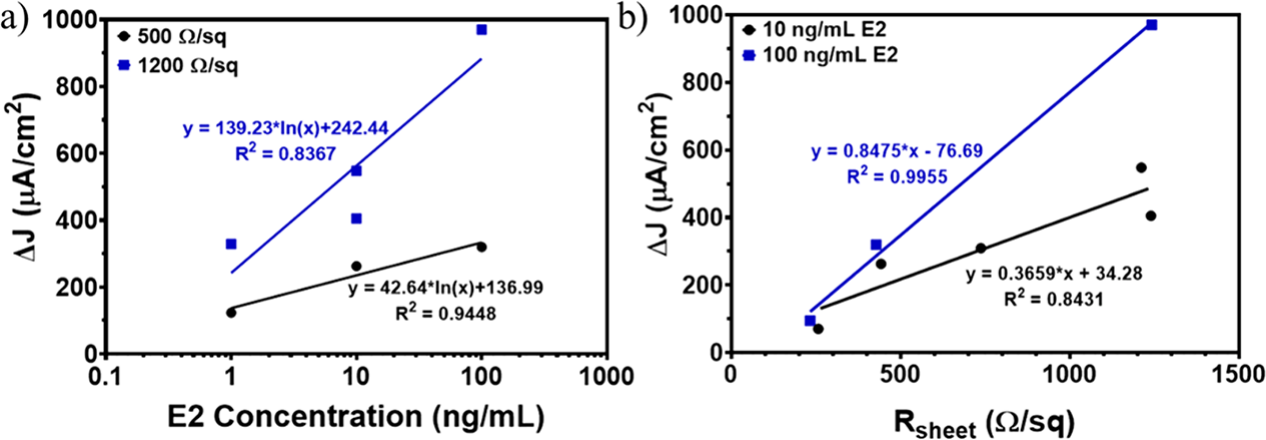
(a) Relationship between current density (Δ*J*) and E2 concentration for biosensors at 500 Ω/sq (black dots) and 1200 Ω/sq (blue squares) showing that Δ*J* increases at different rates for the two different *R*_sheet_ values. The semilogarithmic regression fits and equations at 500 Ω/sq (black solid line) and 1200 Ω/sq (blue solid line) are also shown. (b) The relationship between Δ*J* and *R*_sheet_ for biosensors exposed to 10 ng/mL E2 (black dots) and 100 ng/mL E2 (blue squares) indicates that, for higher *R*_sheet_ values, Δ*J* is higher for the same E2 concentration. The linear regression fits and equations of 10 ng/mL E2 (black solid line) and 100 ng/mL E2 (blue solid line) are also shown.

We quantified the relationship between changes in current density (Δ*J*) calculated using Equation SE.1 presented in the Supporting Information, Section S.5, and E2 concentrations for two different *R*_sheet_ values (Figure 4a). We tested sensors with two different sensor surface sheet resistivities (nominal value of 500 and 1200 Ω/sq, respectively). These results demonstrate a strong positive relationship between current density change and E2 concentration (*R*^2^ ≥ 0.84; Figure 4a; raw data available in Table S1 presented in the Supporting Information, Section S.6). When the biosensor was highly doped (low *R*_sheet_, 250 Ω/sq), the change in Δ*J* was at least 7 times less than that of a low-doped biosensor (high *R*_sheet_, 1200 Ω/sq) for the same E2 concentration (Figure 4b). Two different concentrations of E2, 10 and 100 ng/mL, were exposed to biosensors at varied levels of doping. Linear regression fits were performed between *R*_sheet_ and Δ*J* for the two concentrations (*R*^2^ ≥ 0.84; Figure 4b; raw data available in Table S2 presented in the Supporting Information, Section S.6). The biosensors with *R*_sheet_ of around 1200 Ω/sq exhibited the largest Δ*J* after exposure to E2 as well as the largest current density difference between the 10 and 100 ng/mL concentrations. No data exists for higher *R*_sheet_ values because the biosensors were unable to generate a measurable electrical signal with such low doping levels. Nevertheless, there is a lower limit to doping the Si NWs because extremely low doping prevents the p–n junction from being formed, and the device therefore will not be electrically active. We note that the estrogen levels tested herein are still orders of magnitude higher than the typical natural aquatic conditions; thus, further research to lower the detection limits is needed.

Biosensor sensitivity is known to be affected by the doping of the NWs.^24^ Specifically, when the biosensor surface is highly doped, detection of the target molecule decreases due to the screening effects and the recombination rate.^25^ As the doping concentration of the NWs increases, an electrostatic effect known as the screening effect^26^ occurs within the NWs, where a carrier repels other carriers and creates what is known as a “screening hole” around itself, decreasing the sensitivity of the NWs to surface charge changes that may occur, due to, for example, the introduction of charged molecules (such as the hormones being tested in this work). The electric field within the screening hole is canceled and leads to a lower current density response. Furthermore, as NW doping increases, the carrier recombination rate increases, resulting in a decrease in minority carrier diffusion length and an overall lower current density change.

Our vSiNW-diode biosensor exhibited a substantially higher electrical change compared to traditional hSiNW-FET biosensors that generate electrical current changes at the nanoampere (nA) level.^15^ Such small electrical signals require expensive measurement equipment, such as that reported in ref (10), which employed an expensive current preamplifier (1211, DL Instrument, >$10,000) to collect electrical current changes measured from single hSiNW-FET sensors. For time dependent measurements, the team used a lock-in amplifier (SR830, DSP dual-phase, Stanford Research Systems, >$20,000) to collect the nA sensor signal changes. In comparison, our vSiNW-diode biosensor has electrical current changes in the microamperes (μA) range, demonstrating up to 100 times electrical signal amplification relative to traditional NW biosensors.^27^ This amplification enables the measurement of our sensor signal using inexpensive equipment (∼$200), emphasizing the advantages of our vSiNW-diode biosensors and the potential to use the biosensors in a nonstandard lab setting, such as in water streams for real-time data collection.

### Biosensor Specificity Measurements

**Figure 5:**
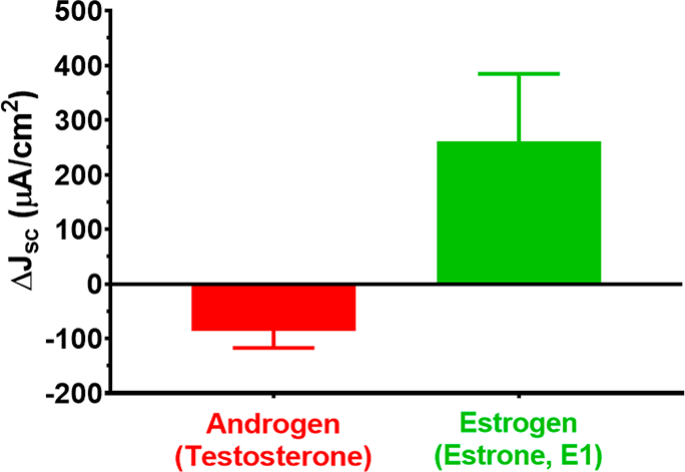
Average short-circuit current density (Δ*J*_sc_) change and standard error (shown as error bars) for the six biosensors, three exposed to testosterone (red bar), and three exposed to estrone (green bar). Each biosensor was exposed to 10 μg/mL of the respective hormone.

We tested detection selectivity of our biosensor using testosterone and E1, two additional hormones, as a positive and negative control, respectively. E1, as another estrogen, was expected to bind to ER-α, and testosterone, as an androgen, was not expected to yield a positive signal change. We confirmed that the hormones were binding only to the receptors (i.e., nothing else on the biosensor surface, which may lead to false positives) and that another estrogenic compound not originally tested generated a positive signal change. The biosensor current density change was negligible when exposed to the testosterone compared to E1 (Figure 5, raw data available in Table S3 presented in the Supporting Information, Section S.6). We also noticed that the current density changes following testosterone exposure exhibited a decrease in current density for all three biosensors, a behavior not observed in biosensors exposed to E1. This is likely because testosterone is known to exhibit negative surface charge^28^ and has a bandgap energy of 5.23 eV.^29^ The excess negative surface charge generated by the testosterone presence will decrease the conductivity of n-type Si NWs resulting in the observed decrease in current density change.

### Biosensor Regeneration Measurements

**Figure 6:**
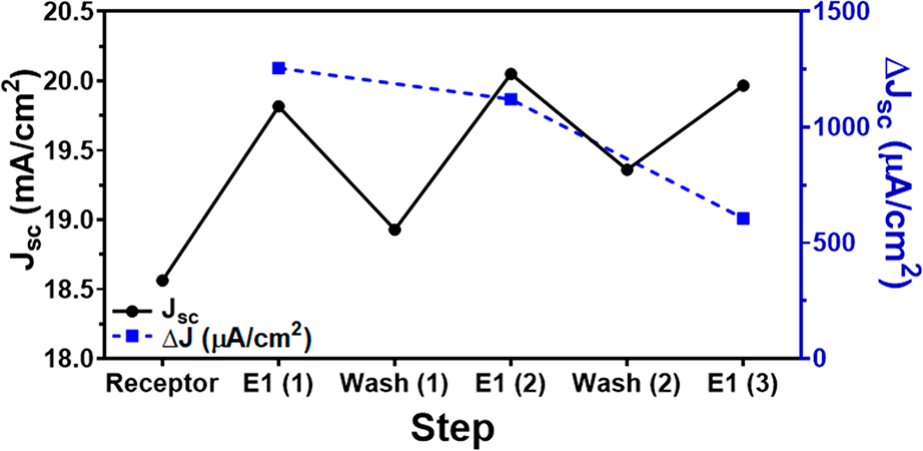
Current density (*J*; left *y*-axis; solid black dots and line) and changes in current density (Δ*J*; right *y*-axis; solid blue squares and dashed line) of one biosensor after three rounds of washing and re-exposure to 10 ng/mL E1.

For field-deployable sensors, demonstration of biosensor reusability is critical. We tested the current density response of the biosensor with exposure to 10 ng/mL E1 followed by 5 min of washing with diluted (0.0001×) phosphate buffer solution (PBS), then re-exposure to E1, *J*–*V*, and another wash and continued this cycle. The current density response of the sensor was within a ± 10% difference for the first two rounds of washing and E1 exposure (Figure 6). During the third round of washing, the biosensor response decreased, possibly due to the damage of the sensor surface from repeated regeneration and functionalization; this observation requires further investigation. Although the vSiNWs could possibly be damaged over the course of the repeated experiments, this appears less likely because the amount of current (*J*) from each incubation remains relatively constant. Another explanation could be that the washing process fails to fully remove the attached E1. Additional testing could involve the biosensor’s performance over greater intervals of time, including characterizing the biosensor shelf life and storage conditions. Previous biosensors have demonstrated that a brief rinse in buffer solution will essentially “reset” the device to its original state, which can be reused for further tests.^30^

## Conclusion

In this work, we present a novel vSiNW-diode biosensor design that is sensitive and selective and can be manufactured using a scalable process. The use of millions of NWs in our design enables sensor measurements that can be performed using inexpensive electrical current sensors and thus outperform hSiNW-FET biosensors. The vSiNW-diode biosensor is a platform technology that can be easily modified to detect multiple species concurrently, and we demonstrated the potential for this design in our initial phase of work presented here. Receptor-based biosensors offer the promise of being able to rapidly monitor endocrine-disrupting compounds in water sources more akin to the perspective of impacted biota than status-quo approaches (i.e., receptor binding rather than mass spectrometry). Receptor-bind biosensors also allow for detection of novel estrogenic metabolites/transformation products that may otherwise evade traditional detection methods. Other nanotechnology approaches integrated with the biosensor could further increase the sensor sensitivity to environmentally relevant detection levels. In addition, with a proper microfluidic design, the metal contacts of the biosensor can be fully protected from the analytes used during the functionalization process, which could enable regeneration of the sensors multiple times without performance degradation over time. The microfluidics will also allow for real-time measurements in a nonlab environment. Our future studies will involve systematically conducting tests with field-collected water samples, to confirm the utility of the reported vSiNW biosensor for field-based water quality monitoring.

## Supporting Information Available

Materials; microfabrication of the Si NW biosensors; biofunctionalization process steps; current density versus voltage (*J*–*V*) measurements; fluorescence measurements; use of aminopolyethylene glycol (Amino PEG) vs ethanolamine to block nonspecific binding; uniform spread of functionalization agents; Δ*J* calculation methodology; chemical structures of E1, E2, and testosterone; and raw data tables for Figures 4a,b and 5 (PDF)
