# Supplementary material for: Sensitive and Specific Detection of Estrogens Featuring Doped Silicon Nanowire Arrays
Source: ACS Omega. 2022 Dec 6;7(50):47341–8. doi: 10.1021/acsomega.1c00210 (PMC9774403; doi:10.1021/acsomega.1c00210)
Supplement: Supplementary file 1 — ao1c00210_si_001.pdf [file ao1c00210_si_001.pdf]

SUPPORTING INFORMATION

Sensitive and Specific Detection of Estrogens Featuring Doped Silicon Nanowire Arrays

Wenqi Duan[†,‡,1#], Hui Zhi[§,¶,2#], Daniel W. Keefe[†,‡], Bingtao Gao[†,‡,3], Gregory H. LeFevre[§,¶*], Fatima Toor[†,‡,*]

[†] Department of Electrical and Computer Engineering, University of Iowa, 205 N. Madison St, Iowa City, Iowa, 52242, USA
[§] Department of Civil and Environmental Engineering, University of Iowa, 4105 Seamans Center, Iowa City, Iowa, 52242, USA
[¶] IIHR-Hydroscience & Engineering, 100 C. Maxwell Stanley Hydraulics Laboratory, Iowa City, IA 52242, United States
[‡]Iowa Technology Institute, University of Iowa, 330 South Madison Street, Iowa City, Iowa, 52242, USA

#Duan and Zhi are equal contribution first co-authors.

---

[1]Current address: Thorlabs, 56 Sparta Avenue, Newton, NJ 07860-2402, USA

[2]Current address: Peking University, College of Urban and Environmental Sciences, Shaw Building No 2, Beijing, Beijing, CN 100871, China

[3]Current address: Zhejiang University, Electronic Science and Technology, Liuhelu 318, Hangzhou, Zhejiang, CN 310058, China

S.1. Materials

3-aminopropyltriethoxysilane (APTES, CAS 13822-56-5), glutaraldehyde solution (50% in H_2O, CAS 111-30-8), estrone (E1, CAS 53-16-7), estradiol (E2, CAS 50-28-2), testosterone (CAS 58-22-0) and phosphate buffer saline (PBS, pH 7.4) were purchased from Sigma-Aldrich and used as received. Alpha estrogen receptor (ER-α) protein were purchased from Thermo Fisher Scientific. All the organic solvents including ethanol, acetone and isopropyl alcohol (IPA) were liquid chromatography-mass spectrometry (LC/MS) grade.

S.2. Nanofabrication process steps for the Si NW biosensor

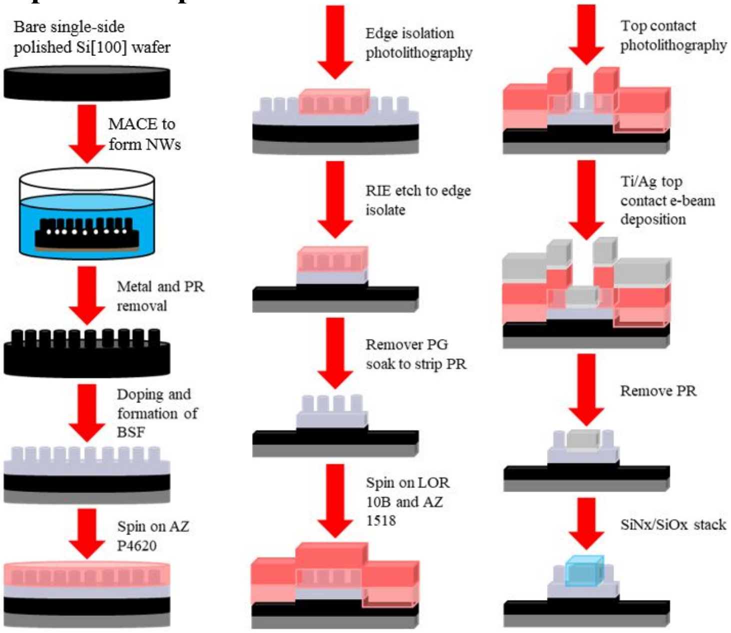

Figure S.1. A visual summary of the nanofabrication process steps for the Si NW biosensor. The front emitter and back surface field (BSF) of the p-n junction are formed concurrently in a rapid thermal annealing furnace. Edge isolation is performed using reactive ion etching (RIE). The top metal contact pattern is exposed using photolithography, followed by a HF clean and then a titanium (Ti) and silver (Ag) deposition using an electron-beam evaporator. The silicon nitride (SiNx)/silicon dioxide (SiOx) stacks are deposited using a sputterer.

The summary schematic representing the nanofabrication process steps are shown in **Figure S.1**. The details of each of the process steps are discussed next.

S.2.1. Fabrication of Si NWs using silver nanoparticles

We purchased single-side polished, two-inch diameter, 280-μm thick, boron-doped (1-10 Ω-cm resistivity), monocrystalline silicon (Si) <100> Czochralski (CZ) wafers from University Wafer. These Si wafers are first thoroughly cleaned using the Radio Corporation of America (RCA) Standard Cleans (SC)[1] and hydrofluoric acid (HF). The RCA SC-1 solution is made of deionized (DI) water, ammonium hydroxide (NH_4OH), and hydrogen peroxide (H_2O_2) in a 5:1:1 ratio. The solution is heated up to and maintained at around 70ºC, and the wafers are submerged for 10 minutes. This basic mixture removes the organic contaminants on the wafer. The wafers are then immersed in a 10 vol% solution of HF to strip the oxide, then rinsed in DI water. RCA SC-2 solution is made using DI water, hydrochloric acid (HCl) and H_2O_2 in a 6:1:1 volume ratio, mixed, and heated to around 70ºC. The wafers are submerged and cleaned in the solution for 10 minutes and finally rinsed in DI water. This removes the remaining ionic residues and slightly passivates the wafer to protect the substrate surface from further contamination.

To prevent the backside of the Si wafer from etching, the back of the Si substrates is coated with photoresist (PR) AZ P4620 and baked for 10 minutes on a hot plate at 120ºC. Another HF clean is performed, and the wafer is rinsed in DI water. To increase the hydrophilicity of the surface and ensure uniform etching, the wafers are submerged in 30 vol% H_2O_2 until the surface is completely oxidized, about a minute or so. A one-step silver (Ag) MACE process is performed by submerging the substrate into a room temperature solution containing silver nitrate ($AgNO_3$), HF, and DI water for 5 minutes. For the target Si NW length of around 500 nm, a 100 mL mixture is composed of 17 mL of HF, 70 mL of DI water, and 13 mL of 154 mM $AgNO_3$, resulting in a 5M HF and 20 mM $AgNO_3$ concentration solution. The solution is stirred continuously as the etching occurs. After etching, the backside PR is removed with an acetone and isopropanol (IPA) rinse, followed by a dip in DI water. The residual Ag nanoparticles at the bottom of the Si NWs, are removed by submerging the substrate in room temperature nitric acid (HNO_3) for 3 minutes and rinsed in DI water again. The Si NW samples are then immersed in a buffered oxide etchant (BOE) for 2 minutes to further clean the samples, rinsed in DI water and gently dried using a dry nitrogen (N_2) gun.

S.2.2. Simultaneous front doping and back-surface-field formation

A nanofabrication process was developed by our team to simultaneously achieve frontside n+ doping and p+ back-surface-field (BSF) formation. The frontside n+ emitter is formed by proximity doping using ammonium dihydrogen phosphate (ADP)[2] as the spin-on-dopant. ADP solution is prepared by mixing 0.85 wt% ADP and DI water. The source wafers are prepared for proximity doping: they are first cleaned in RCA SC-2 cleaning solution, then the oxide is removed with a dip in HF solution. Finally, the source wafer is dipped in Nanostrip solution for 10 minutes at room temperature to increase the hydrophilicity of the surface by forming a dense oxide film on the surface to help the ADP solution spread uniformly across it. The ADP solution is then spin-coated on the polished side of the Si source wafer at 2000 rotations per minute (rpm) with a 200 rpm/s ramp for 1 minute using a Laurell Technologies Spin Coater and baked for 8 minutes at 100°C on a hot plate.

The backside is coated with a thin layer of aluminum (Al) paste that is carefully applied, using a scraper as a squeegee to clean off the excess and pre-baked at 180°C for 5 minutes on a hot plate. Al is a known p-type dopant of Si; therefore the presence of Al paste ensures a robust p+ BSF. The Al also serves as the back contact of the biosensor. The final setup is then assembled on a quartz plate to be inserted into an MTI Corporation EQ-RTP-1000D4, a rapid thermal annealing (RTA) furnace. The Si source wafer is placed ADP side up on the quartz plate, while the Si substrate is placed NW side down, separated by 500 μm Si spacers. This setup is then placed into the RTA chamber, which is then pumped down. N_2 gas is then vented into the chamber at a 100 sccm flow, and the pump is adjusted so that the pressure in the chamber is maintained at 1 atm (760 Torr). The chamber temperature is ramped up to 950°C in 10 minutes and maintained at that temperature for 10 minutes. This process results roughly in a front junction depth of 0.7 μm,[3] which is deeper than the NW length, and ensures that the junction is formed under the NWs. Afterwards, the sample is removed once the chamber has naturally cooled to room temperature. The phosphosilicate glass (PSG) residue that is formed on the surface of the doped samples is removed by a 30 second dip in BOE. The final R_{sheet} of the Si NW samples is then measured using a Signatone S-302 4-point Resistivity Probe in our lab.

S.2.3. Edge isolation

To prevent shorting out the sensor, edge isolation is performed on each p-n junction device. PR AZ P4620 is spin-coated on the front at 4000 rpm with a 4000 rpm/s ramp for 1 minute and dried on a hot plate at 100°C for 4 minutes. Since the developer will etch Al, the backside is also coated in a layer of AZ P4620. Since AZ P4620 is a thick resist, there may be buildup on the edges of the sample where the photoresist is more elevated than the rest of the surface, called edge beads. This can behave as an unwanted spacer between the substrate and the mask, as well as lead to cracking or damaging the substrate. To remove any edge beads that have formed, a cotton swab dipped in acetone is used to smooth out the edges.

To ensure that the PR has sufficient water content when photolithography is performed, the samples are left on the wet bench for 20 minutes. Rehydration is an important step to guarantee a high development rate. At this step, the whole wafer is cleaved into quarters in anticipation for one sensor per quarter wafer. Afterward, the samples are then exposed to the pattern on an OAI Mask Aligner Model 800 for 24 seconds. The substrate is developed in AZ400K developer 1:4 developer for at least 4 minutes. The exposed pattern is an 11.5 cm by 11.5 cm square in the middle of the Si substrate.

The excess PR is rinsed off with more AZ 400K 1:4 and DI water to guarantee a clean edge. The sample is then etched by reactive ion etching (RIE) of Si using an Oxford Instruments RIE NGP80 machine. The dry etching is performed for 1 minute using a combination of tetrafluoromethane (CF_4), O_2 and Ar gases. After etching, the PR is stripped by soaking in MicroChem Remover PG heated to 80°C for 5 to 15 minutes until the surface is clean. The samples are then rinsed in IPA and gently dried with an N_2 gun. A final R_{sheet} value is measured before front contacts are created.

S.2.4. Top contact patterning and metal deposition

Top contact patterning is done by first spin coating on two PRs. A lift-off resist (LOR) 20B is first spun on at 3000 rpm for 1 minute to make the PR easier to remove after metal deposition and dried on a hot plate at 150°C for 4 minutes. The backside is again coated in AZ P4620 and dried using the same instructions as mentioned previously. The front side is then coated with a layer of AZ 1518, spun on at 3500 rpm for 1 minute and dried on a hot plate at 100°C for 3 minutes. The sample is exposed for 8 seconds to the contact pattern using photolithography and submerged in AZ 400K 1:4 developer.

To facilitate smoother metal deposition, oxide removal of the exposed Si area is then performed by dipping the samples for 30 seconds in BOE. The samples are thoroughly dried under an N_2 spray. Using an Angstrom Engineering 6-pocket E-Beam Evaporator, 50 nm of Ti and 1 μm of Ag are deposited to form a top metal contact. To lift off the PR, the samples are then submerged in 80°C Remover PG while stirred and left overnight if necessary due to the thickness of the metal. After the samples are removed from the Remover PG, the samples are rinsed in IPA and DI water to ensure the removal of any remaining PR.

S.2.5. Dielectric protection of top metal contact

To protect the front metal contact during future wet biofunctionalization process steps, two stacks of alternating silicon nitride (SiNx) and silicon dioxide (SiO_2) layers are deposited on the front surface. The dielectric pattern is designed to leave an open area in the center of the surface for the exposed NWs. The Si substrate are spin-coated with PR using the same layered process for

top contact patterning. This time, the sample is exposed to a different mask that ensures everything except for the center of the sensor and the contact pad will be covered by the dielectrics. The samples are inserted into an IntlVac - Nanochrome I Sputterer, where 50 nm of SiOx and 100 nm of SiNx are deposited alternately until two layers of each dielectric are placed down. We selected the dielectric stack thickness to ensure around 10^{-5} g/m²/day water vapor transmission rate (WVTR), which was found to be sufficiently low to form a barrier of protection against liquids that may loosen the metal contact from the Si surface and render the sensor non-functional. The PR is lifted off, after the dielectric deposition, by submerging the substrates in 80°C Remover PG overnight. The fabricated sensors are then washed with IPA and DI water, dried with N_2 gun, and ready for biofunctionalization.

S.3. Biofunctionalization of the Si NW biosensor for hormone detection

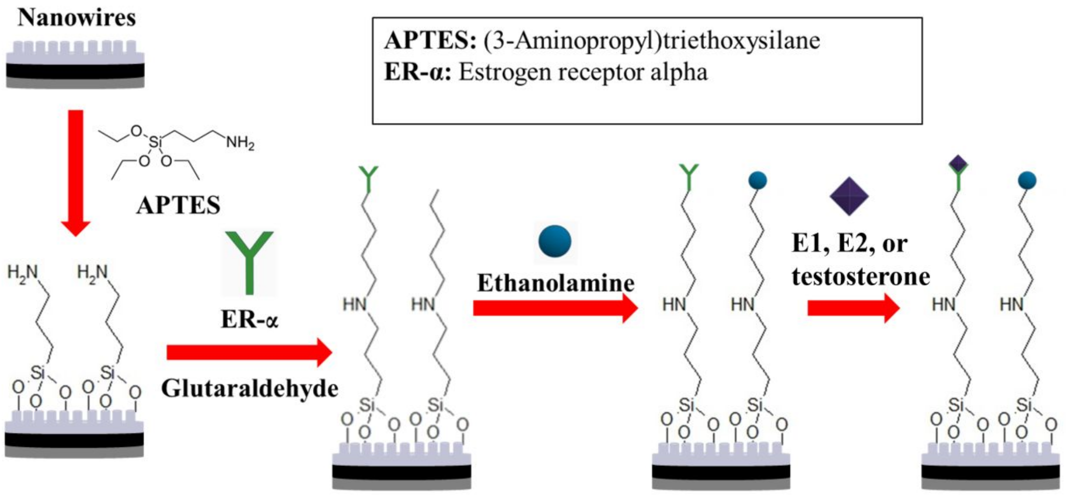

Figure S.2. A visual summary of the biofunctionalization steps of Si NW biosensor utilized to detect hormones. The nanofabricated Si NW biosensor is functionalized using (3-aminopropyl) triethoxysilane (APTES), followed by glutaraldehyde. This bifunctional linker allows for the immobilization of the estrogen receptor-alpha (ER-α) on the Si NW surface. The ER-functionalized surface was then passivated with ethanolamine to minimize unspecific binding. Next the target hormone species was introduced on the Si NW biosensor surface for detection.

The summary schematic representing the biofunctionalization steps are shown in **Figure S.2**. The details of each of the process steps are discussed next.

S.3.1. Sensor biofunctionalization chemistry and process details

The Si NW biosensor surface is first functionalized using 10 mL 2% (3-Aminopropyl)triethoxysilane (APTES) solution in ethanol/H_2O (95/5, v/v) for 2 hours, then the surface is rinsed with ethanol, and dried using a N_2 gun. Next, the biosensor surface is functionalized using 10 mL 2.5% glutaraldehyde solution in DI water for 1 hour, rinsed with DI water, and dried via a N_2 gun. This bifunctional linker contains two aldehyde terminals, which enables one end to bind to the amine-terminated APTES and the other end to immobilize the ER-α protein. Following drying, the ER-α protein is covalently bound onto the surface of the Si NWs by incubating the sensor in 2 mL 10 µg/mL ER-α protein for 6 hours. The unbound ER-α protein is then removed with a 0.01X PBS buffer wash and the surface is dried with N_2. The estrogen receptor-functionalized surface is then passivated with 10 mL 100 mM ethanolamine in 0.01X PBS buffer to minimize non-specific binding for 1 hour, followed by drying with N_2.

Following functionalization with ER-α, we tested the biosensors with three different hormones: estrone (E1) and 17β-estradiol (E2), and testosterone (a non-estrogen androgen hormone that should not bind to ER-α) at concentrations of 1–10,000 ng/mL. **Figure S.3** shows the stereochemical structures of the three hormones tested with our biosensor. 5 mL 0.01X PBS buffer containing target hormone is introduced onto the post-receptor Si NW surface, and the sensor is incubated for 1 hour. After incubation, the surface is dried with N_2 gun and ready for electrical current measurements. All the steps are performed on an orbital shaker table (Cole-Parmer) to ensure uniform coverage of the functionalization agents on the Si NW sensor surface. To regenerate the biosensor, the biosensor surface is washed with pure buffer solution (0.0001X PBS buffer) for 5 minutes.

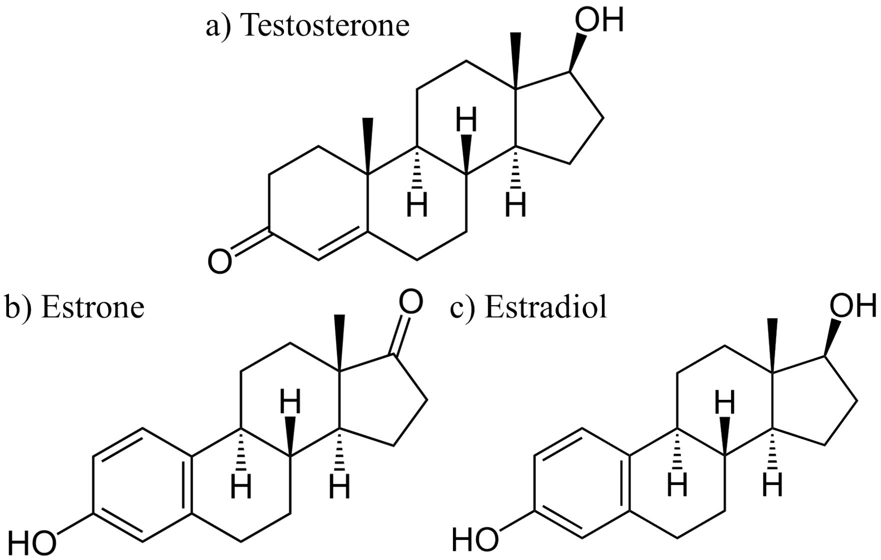

Figure S.3. Stereochemical structures of a) testosterone, b) estrone (E1), and c) estradiol (E2).

S.3.2. Use of aminopolyethylene glycol (Amino PEG) versus ethanolamine to block non-specific binding

After functionalization, there is no guarantee that every amine has an estrogen receptor attached to its end. Therefore, a molecule is introduced in the biofunctionalization process to block all the unlinked amines. In our initial experiments, we passivated the unlinked amines with a reagent called methoxypolyethylene glycol amine (amino PEG) as suggested in Ref. [4]. The molecular weight of amino PEG depends on the number of polyethylene glycol (PEG) molecules conjugated together. However, even the smallest iteration of one PEG molecule is far too large for our sensor surface and possibly interfered with the receptor binding. We found this because, after the sensor incubation in amino PEG, we measured a significant degradation in the effectiveness of the biosensor, as seen in **Figure S.4(a)**. Therefore, to prevent the biosensor response from degrading after functionalization, we used ethanolamine as an alternative passivant, as both a smaller molecule and an inexpensive alternative. This switch significantly reduced the degradation of current density-voltage (J-V) response of the biosensor, as shown in **Figure S.4(b)**, where after amino PEG functionalization the current density dropped by around 2 mA/cm^2, while with ethanolamine the drop was around 0.6 mA/cm^2.

It is important to note that **Figure S.4** shows light-based J-V curves since they were collected at a time in the biosensor development where the team was optimizing the sensor

performance metrics. The concentration dependent response J-V data in the manuscript is based on dark measurements that eliminate variations in the sensor current density which may be caused by variable light intensity

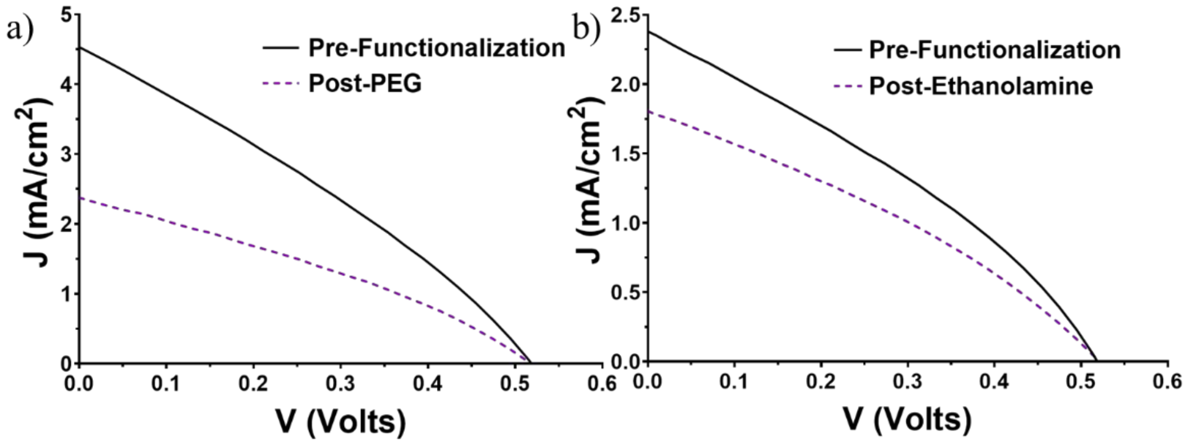

Figure S.4. JV curves before functionalization (black solid line) and after using a) amino PEG (purple dashed line), showing the strong degradation that occurred in the biosensor versus b) ethanolamine (purple dashed line), where the degradation is significantly less.

S.3.3 Fluorescence imaging to confirm uniform distribution of receptor on the biosensor surface

We tested the Si NW biosensor surface with fluorescence imaging to confirm uniform distribution of the biofunctionalized ER-α receptor over the surface. For preparing the sensor for fluorescence imaging, the biosensor was functionalized up to the ER-α receptor as explained in Section S.3.1., the Si NW surface was stained with ER-α protein monoclonal antibody (Thermo Fisher product # MA5-13191) at a dilution of 1:750 in 0.01X PBS buffer solution overnight at 4 °C. The sample was then incubated with DyLight 488-conjugated goat anti-mouse IgG secondary antibody (Thermo Fisher product # 35502) at a dilution of 1:500 in 0.01X PBS buffer solution for 1 hour at room temperature, then the sample surface was dried using a N_2 gun. All fluorescence data were collected on the Zeiss LSM 710 confocal microscope.

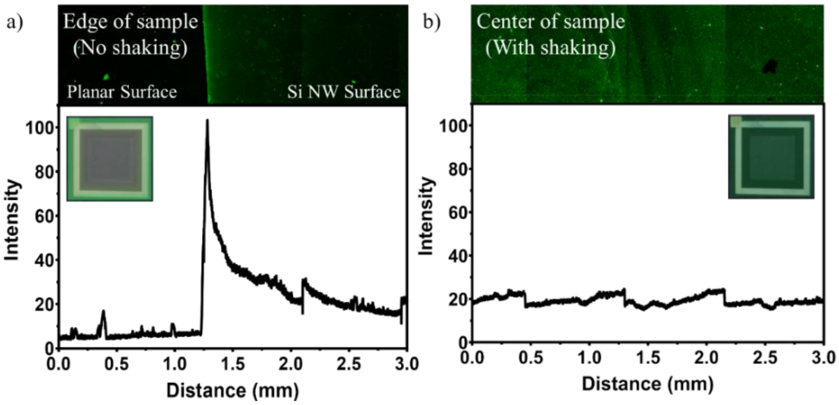

Figure S.5. Fluorescence data taken using a confocal microscope showing a) all the receptors clustered around the edge of the biosensor when no shaking was used, and b) significant improvement of uniformity of the receptors on the surface of the biosensor due to the use of a shaking table. The insets demonstrating the distribution of the receptors on the surface.

As shown in **Figure S.5(a)**, receptors did not spread uniformly on the surface when the sample was not agitated during functionalization. The sharp increase in intensity shows all the receptors clustered at the edges of the samples and very little of the receptors were present in the center of the sample, which could result in decreased the responsivity of the sensor. If the receptors were not spread evenly over the surface, they will compete for the hormones and therefore the sensor signal change will be lower. To resolve this, a shaking table was utilized to slowly shake the beaker holding the biosensor while it was immersed in the receptor solution. This helped to evenly distribute the receptors across the sensor surface. After being stained by fluorescent antibody, the receptors are much more evenly distributed, as shown in **Figure S.5(b)**. The entire sensor surface is at the same fluorescent intensity instead of the high intensity spike seen on the edge of the sensor in the no shaking case.

S.4. Current density versus voltage (J-V) measurements

The current versus voltage (I-V) measured were performed using a Keithley 2400 source meter. The front of the biosensor was contacted with a tungsten needle attached to a Quater XYZ 300 TL micro positioner, and the back of the sensor sat on an Oriel Instruments Basic PVIV vacuum chuck. Both the front and back contacts of the biosensor were then connected through cables to the source meter. A PC LabView program processed the I-V data measured by the source meter connected to the biosensor. The electrical current values were converted to current density (J), using the biosensor active area. J-V curves were measured in the dark before and after the exposure of E2 to eliminate any external influences on the biosensor. For consistency, the J value at 600 mV was selected so that the bias voltage was high enough that the biosensor was effectively “turned on” and sufficient electrical current was flowing through the device.

S.5. ΔJ calculation methodology

After the biosensor is functionalized with the receptor, a JV curve is first taken as a baseline. After the introduction of estrogen or any other hormone to the biosensor, another JV curve is taken and compared to the baseline data. Using these two curves, a change in current density (ΔJ) is calculated:

$$\Delta J = J_{Post\text{ - }Estrogen} - J_{Post\text{ - }ER\alpha}. \quad \text{(SE.1)}$$

S.6. Raw data for Figures 4(a), 4(b), and 5

Sample #	Concentration of E2 (ng/mL)	ΔJ (μA/cm^2) for 500 Ω/sq	ΔJ (μA/cm^2) for 12000 Ω/sq	Measured R_{sheet} (Ω/sq)
**WD14_B01**	10		405.14	1240
**WD14_B02**	100		970.13	1243
**WD14_B04**	10		547.90	1212
**WD16_B03**	1		328.95	1074
**WD15_B02**	10	262.59		443

**WD15_B04**	100	319.65		429
**WD16_B05**	1	123.28		574

Table S.1. Figure 4(a) raw data table of E2 concentration, corresponding measured ΔJ (μA/cm^2) and R_{sheet} (Ω/sq) values for biosensors fabricated at two different nominal R_{sheet} values of 500 Ω/sq and 1200 Ω/sq.

**Sample #**	**ΔJ (μA/cm^2) for 10 ng/mL E2**	**ΔJ (μA/cm^2) for 100 ng/mL E2**	**Measured R_{sheet} (Ω/sq)**
**WD14_B01**	405.14		1240
**WD14_B04**	547.90		1212
**WD14_B08**	70.32		257
**WD15_B02**	262.59		443
**WD16_B08**	308.99		738
**WD14_B02**		970.13	1243
**WD14_B07**		94.52	233
**WD15_B04**		319.65	429

Table S.2. Figure 4(b) raw data table of measured R_{sheet} (Ω/sq) values and ΔJ (μA/cm^2) response of biosensors exposed to two different E2 concentrations, 10 ng/mL and 100 ng/mL.

	**ΔJ_{sc} (μA/cm^2)**	
	**Androgen (10 μg/mL)**	**E1 (10 μg/mL)**
**Average**	-86.10	261.34
**Standard Deviation**	53.52	213.56

Table S.3. Figure 5 raw data table of comparison of ΔJ_{sc} of six biosensors from the same batch, three exposed to E1 and three to androgen, both in the same concentration, 10 μg/mL. The average ΔJ and standard deviation for the three biosensors in each category are calculated.

AUTHOR INFORMATION

Corresponding Authors

*FT: fatima-toor@uiowa.edu *GHL: gregory-lefevre@uiowa.edu

Author Contributions

#These authors contributed equally as co-first authors. The manuscript was written through contributions of all authors. All authors have given approval to the final version of the manuscript.

References

(1) Kern, W. The Evolution of Silicon Wafer Cleaning Technology. *Journal of the Electrochemical Society* **1990**, *137* (6).

(2) Tang, Y. H.; Zhou, C. L.; Wang, W. J.; Zhao, Y.; Zhou, S.; Fei, J. M.; Cao, H. B. N+ emitters realized using Ammonium Dihydrogen Phosphate for silicon solar cells. *Solar Energy* **2013**, *95*, 265-270. DOI: 10.1016/j.solener.2013.04.024.

(3) Duan, W.; Gao, B.; Haque, K. A. S. M. E.; Toor, F. Performance enhancement techniques for the front and back of nanostructured "black silicon" solar cells. *Journal of Photonics for Energy* **2018**, *8* (3), 034001.
(4) Zhang, G.-J.; Huang, M. J.; Luo, Z. H. H.; Tay, G. K. I.; Lim, E.-J. A.; Liu, E. T.; Thomsen, J. S. Highly sensitive and reversible silicon nanowire biosensor to study nuclear hormone receptor protein and response element DNA interactions. *Biosensors and Bioelectronics* **2010**, *26* (2), 365-370.
